# Supplementary material for: Neuronal expression of S100B triggered by oligomeric Aβ peptide contributes to protection against cytoskeletal damage and synaptic loss
Source: Front Mol Neurosci. 2025 Aug 8;18:1636365. doi: 10.3389/fnmol.2025.1636365 (PMC12370693; doi:10.3389/fnmol.2025.1636365)
Supplement: Supplementary file 1 [file Data_Sheet_1.docx]

Supplementary Material

Neuronal expression of S100B triggered by oligomeric Aβ peptide contributes to protection against cytoskeletal damage and synaptic loss

Joana Saavedra^1,2,3^, Mariana Nascimento^1,2,4^, António J. Figueira^5,6^, Marina I. Oliveira da Silva^1,2^, Tiago Gião^1,2,3^, João Oliveira^1,2,7^, Márcia A. Liz^1,2,8,9,10^, Cláudio M. Gomes^5,6^, Isabel Cardoso^1,2,3,*^

^1^i3S - Instituto de Investigação e Inovação em Saúde, Universidade do Porto, 4200-135, Porto, Portugal

^2^IBMC - Instituto de Biologia Molecular e Celular, Universidade do Porto, 4200-135, Porto, Portugal

^3^ICBAS - Instituto de Ciências Biomédicas Abel Salazar, Universidade do Porto, 4200-135, Portugal

^4^FMUP- Faculdade de Medicina, Universidade do Porto, Portugal

^5^BioISI-Instituto de Biosistemas e Ciências Integrativas, Faculdade de Ciências, Universidade de Lisboa, 1749-016, Lisboa, Portugal

^6^Departamento de Química e Bioquímica, Faculdade de Ciências, Universidade de Lisboa, 1749-016 Lisboa, Portugal

^7^FCUP- Faculdade de Ciências, Universidade do Porto, 4169-007, Porto, Portugal

^8^UMIB – Unit for Multidisciplinary Research in Biomedicine, ICBAS – School of Medicine and Biomedical Sciences, University of Porto, 4050-313 Porto, Portugal

^9^ITR – Laboratory for Integrative and Translational Research in Population Health, 4050-600 Porto, Portugal

^10^Molecular Pathology and Immunology Department, ICBAS – School of Medicine and Biomedical Sciences, University of Porto, 4050-313 Porto, Portugal

*** Correspondence:**Isabel Cardoso
icardoso@ibmc.up.pt

# Material and methods

## Cofilin-actin rods immunohistochemistry

Free-floating 30 μm-thick coronal mice (AβPPswe/PS1A246E, Alzheimer’s disease mouse model) brain sections, obtained with the cryostat, were permeabilized with -20ºC methanol, for 1 minute, and then change to fresh -20 ºC methanol for another 5 minutes. Next, tissues were rinsed 3x with PBS, and then blocked with 10% donkey serum in PBS containing 1% BSA, for 1 hour at RT. This was followed by incubation with primary rabbit anti-cofilin antibody (D3F9; 1:2000; Cell Signaling), diluted in 1% BSA in PBS, ON at 4ºC. On the next day, tissues were washed with PBS (6x, 10 minutes) and incubated with Alexa Fluor-568 goat anti-rabbit IgG antibody (A11011; 1:500; Invitrogen), in 1% BSA in PBS, for 2 hours at RT. Then tissues were washed with PBS (6x, 10 minutes) and incubated with DAPI in PBS (1:100; Bio-Rad) for 15 minutes at RT. After, tissues were washed in 70% ethanol for 5 minutes, and after removing the ethanol, with 0,1% Sudan Black in 70% ethanol for 10 minutes. Finally, tissues were washed with 70% ethanol for 20-30 seconds (2x), and then once with PBS for 15 seconds. All of the previous steps were performed with agitation. The brain sections were then mounted on normafrost slides silane pre-coated (Normax), with ibidi mounting media (without DAPI). The tissues were visualized and photographed using a Leica DMI6000 FFW microscope equipped with a Hamamatsu FLASH 4.0 camera (Hamamatsu, Japan), and the LAS X software, and pictures were taken using a 20x magnification. Number of cofilin-actin rods per area was counted using Fiji software [1]. Negative controls were prepared in the absence of primary antibodie and showed no significant signal (data not shown).

## Proteins and peptides

Human myc-tagged (TRTRPL**EQKLISEEDL**AANDILDYKDDDDKV) S100B was expressed in *E. coli* (BL21 (DE3) E. Cline Express, Lucigen) and purified to homogeneity as described ^42,43^. S100B-myc concentrations were estimated as homodimer equivalents using by UV spectroscopy at 280 nm using the theoretical extinction coefficient value of ε_280 nm_ = 5,960 M^-1^cm^-1^

## S100B internalization by neurons

Primary neurons (DIV 7) were treated during 30 minutes with S100B myc tag (2.5 μM) and then fixed with 4% PFA in 1x cytoskeleton preservation buffer (10mM MES pH 6.1; 138mM KCl; 3mM MgCl_2_; 2mM EGTA pH 7; 0.32M sucrose), for 30 minutes at RT. After fixation, cells were washed with PBS (3x, 5 minutes) and permeabilized with 0,1% Triton X-100 in PBS for 10 minutes at RT. Cells were, once again, washed with PBS (3x, 5 minutes) and blocked with 5% BSA in PBS for 1 hour, then incubated with primary mouse MYC tag 4A6 antibody (05-724; 1:500; Merck Millipore) and rabbit βIII tubulin (302302; 1:1000; Synaptic Systems) in 1% BSA in PBS, ON at 4 ºC. On the next day, cells were washed with PBS (3x, 5 minutes) and incubated with Alexa Fluor-488 goat anti-mouse IgG antibody (A11029;1:1000; Invitrogen) and Alexa Fluor-568 goat anti-rabbit IgG antibody (A11011; 1:1500; Invitrogen) with 1% BSA in PBS for 1 hour at RT, and then washed once again with PBS (3x, 5 minutes) After this, coverslips were mounted with FluoroshieldTM with DAPI (Sigma-Aldrich). Cell visualization and image capture were done using the Zeiss Axio Imager Z1 microscope equipped with an Axiocam MR3.0 camera and Axivision 4.9.1 software, with a 40x magnification. Negative controls were prepared in the absence of primary antibodies and showed no significant signal (data not shown).

## S100B expression by neurons

Primary neurons (DIV 7) were fixed with 4% PFA in 1x cytoskeleton preservation buffer (10mM MES pH 6.1; 138mM KCl; 3mM MgCl_2_; 2mM EGTA pH 7; 0.32M sucrose), for 30 minutes at RT. After fixation, cells were rinsed 3x with PBS, and then permeabilized with 0,3% Triton X-100 in PBS for 15 minutes, at RT. Next, cells were rinsed 3x with PBS again, and blocked with BSA 5% in PBS for 1 hour, at RT. After blocking, neurons were incubated with primary mouse anti-βIII tubulin antibody (1:2000; Promega) and with primary rabbit anti-S100B antibody (1:200; Abcam), in BSA 1% in PBS, ON at 4 ºC. On the next day, cells were rinsed 3x with PBS, and incubated with Alexa Fluor-488 goat anti-mouse IgG antibody (1:1500; Invitrogen) and Alexa Fluor-568 goat anti-rabbit IgG antibody (1:1500; Invitrogen) at 1% BSA in PBS for 1 hour at RT. After this, cells were rinsed 3x with PBS and coverslips were mounted with FluoroshieldTM with DAPI (Sigma-Aldrich). Cell visualization and image capture was done using the Zeiss Axio Imager Z1 microscope equipped with an Axiocam MR3.0 camera and Axivision 4.9.1 software, with a 40x magnification. Negative controls were prepared in the absence of primary antibodies and showed no significant signal (data not shown).

## Cofilin and βIII tubulin immunocytochemistry in primary rat neurons

DIV5 rat neurons were incubated with 10 μM Aβ42 oligomers for 24 or 48 hours or at DIV6 with 1.0, 2.5, 5.0, 10.0 μM S100B for 24 hours; S100B was added halfway through the 48-hour Aβ42 treatment. Neurons were fixed at DIV 7 with 4% PFA in 1x cytoskeleton preservation buffer (10mM MES pH 6.1; 138mM KCl; 3mM MgCl_2_; 2mM EGTA pH 7; 0.32M sucrose), for 30 minutes at RT, and then rinsed 3x times with PBS, and incubated with -20ºC methanol, for 3 minutes at RT. Next, cells were again rinsed 3x with PBS and incubated with blocking solution (2,5% donkey serum in PBS containing 1% BSA (Bovine serum albumin), for 1 hour at RT. This was followed by incubation with primary rabbit anti-cofilin antibody (D3F9; 1:2000; Cell Signaling) and mouse anti-βIII tubulin antibody (G712A; 1:2000; Promega), diluted in 1% BSA in PBS, overnight (ON) at 4ºC. On the next day, neurons were rinsed 3x with PBS and incubated with Alexa Fluor-568 goat anti-rabbit IgG antibody (A11011; 1:1000; Invitrogen) and Alexa Fluor-488 goat anti-mouse IgG antibody (A11029;1:1000; Invitrogen), in 1% BSA in PBS, for 1 hour at RT. After incubation, neurons were rinsed 3x with PBS and coverslips were mounted with FluoroshieldTM with DAPI (Sigma-Aldrich). Cell visualization and image capture was done using the Zeiss Axio Imager Z1 microscope equipped with an Axiocam MR3.0 camera and Axivision 4.9.1 software, with a 40x magnification. Finally, cells with cofilin-actin rods were counted and divided per total number of cells. Negative controls were prepared in the absence of primary antibodies and showed no significant signal (data not shown).

# Supplementary Figures


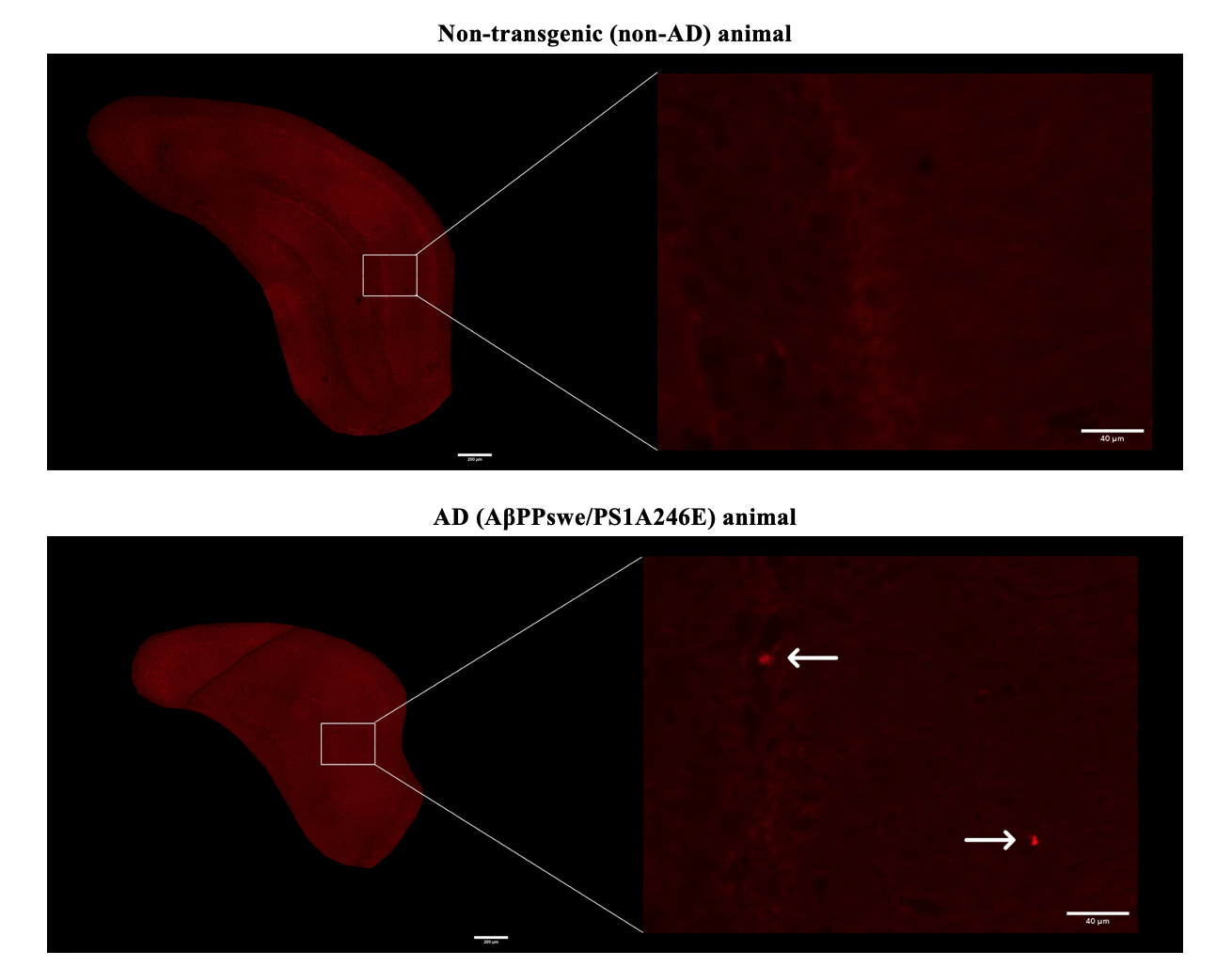


**Supplementary Figure 1. Cofilin-actin rod formation in hippocampus of NT and AD mice.** Cofilin-actin rod formation (arrows) in the hippocampus of AD (AβPPswe/PS1A246E) and non-transgenic (non-AD) animals, at 7 months. Red: Cofilin. Hippocampus Scale bar = 200 μm; Enlarged insertion, scale bar = 40 μm.


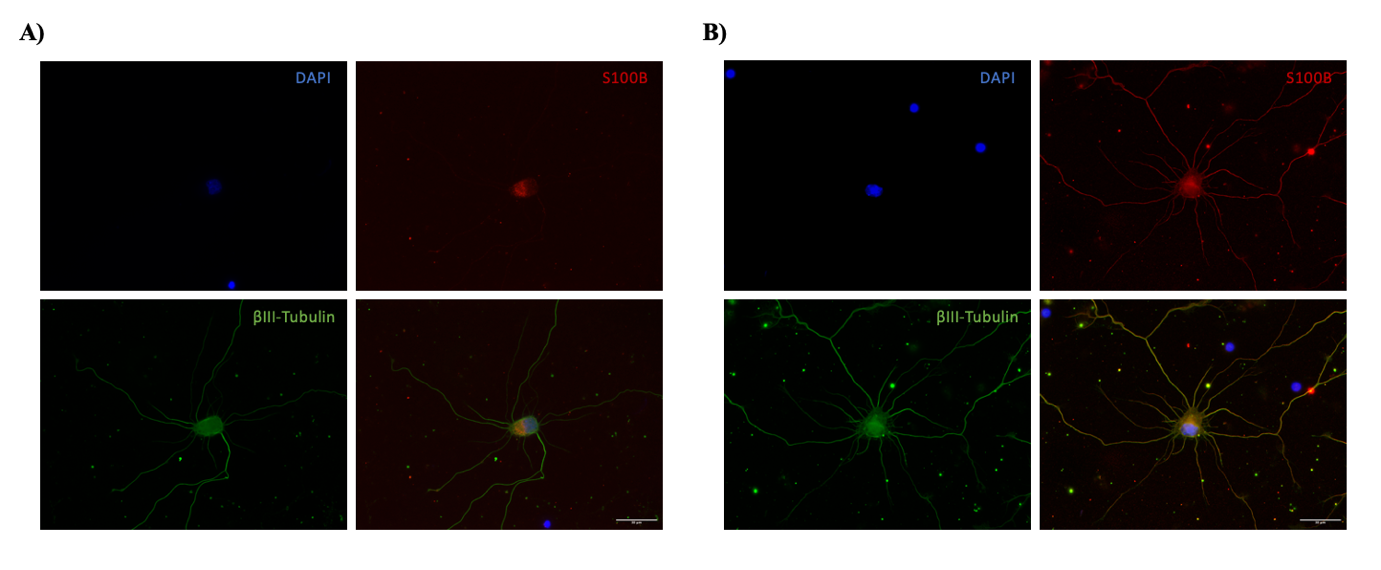


**Supplementary Figure 2.** S100B expression and internalization in primary cultures of rat hippocampal neurons. Endogenous expression **(A)** and exogenous internalization **(B)** of S100B in rat hippocampal neurons (DIV7). Green: βIII-Tubulin; Red: S100B. Blue: DAPI. Scale bar = 30 μm.


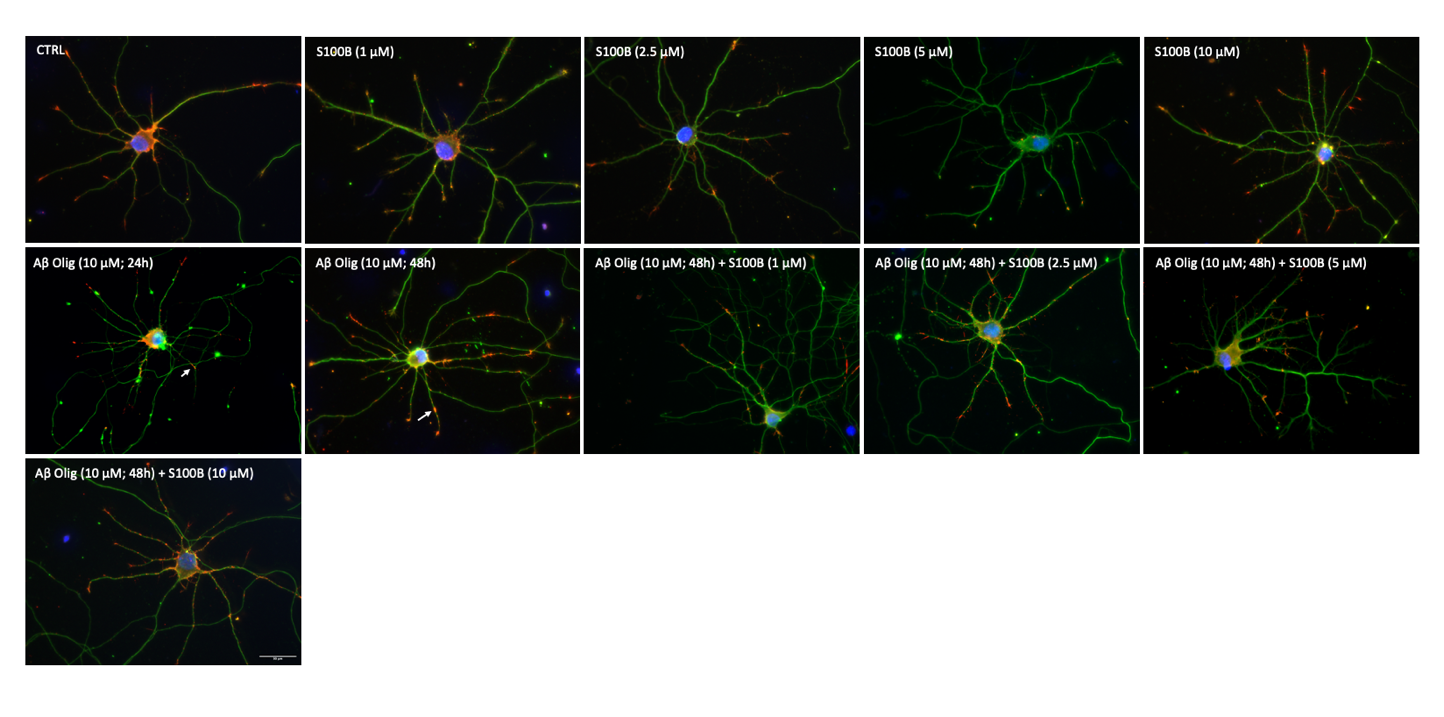


**
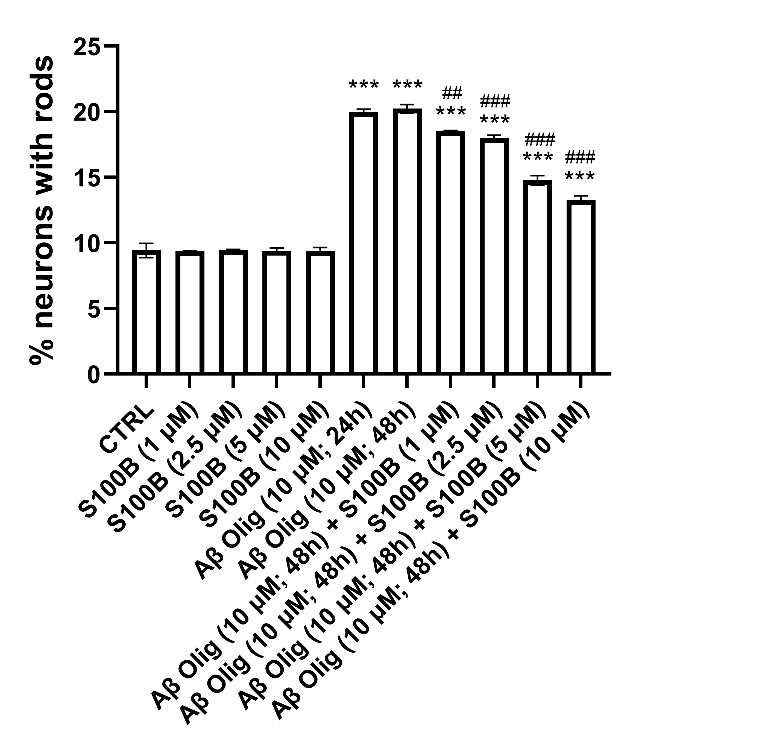
**

**Supplementary Figure 3. Impact of S100B on Aβ oligomers-induced cofilin-actin rod formation in primary cultures of rat hippocampal neurons.** Representative images and quantification plot of rat hippocampal neurons (DIV7) showing increased % of cells with cofilin-actin rods after incubation with Aβ oligomers (Aβ Olig) 24h or 48h (10 μM). S100B (1; 2.5; 5; 10 μM) added after 24 hours partially rescued the Aβ-induced phenotype in a dose dependent manner. S100B alone did not produce alterations, as compared to control (CTRL). White arrows point out cofilin-actin rods. Green: βIII-Tubulin; Red: Cofilin. Blue: DAPI. Scale bar = 30 μm. Data are expressed as mean ± SEM. * or ^#^ p<0.05; *** or ^###^ p<0.001. (* comparation with control; ^#^ comparation with Aβ oligomers 48h).
